# Health-Related Quality of Life among Women Breast Cancer Patients in Eastern China

**DOI:** 10.1155/2018/1452635

**Published:** 2018-07-03

**Authors:** Qing Chen, Shunping Li, Min Wang, Liu Liu, Gang Chen

**Affiliations:** ^1^School of Health Care Management, Shandong University, Jinan 250012, China; ^2^Key Laboratory of Health Economics and Policy Research, NHFPC, Shandong University, Jinan 250012, China; ^3^Qingdao Municipal Hospital, Qingdao 266011, China; ^4^Flinders Centre for Innovation in Cancer, Flinders University, Adelaide, SA 5042, Australia

## Abstract

**Objectives:**

Breast cancer is one of the major cancers in Chinese women. European Organization for Research and Treatment of Cancer Quality of Life Questionnaires (EORTC QLQ-C30 and QLQ-BR23) are now the most common and well developed instruments assessing the health-related quality of life (HRQOL) of breast cancer patients internationally, whereas there are relatively few Chinese studies. This study has two aims: to investigate the HRQOL and explore which dimensions of HRQOL play more important roles in breast cancer patients' overall quality of life in China and to explore the latent factor structure and the potential complementary relationship between these two EORTC questionnaires.

**Methods:**

This cross-sectional and descriptive study was performed from October 2014 to February 2015 in Qingdao Municipal Hospital, China. A total of 621 women breast cancer patients were enrolled. EOTRC QLQ-C30 and QLQ-BR23 were used to evaluate the HRQOL of the participants. The nonparametric test, multiple linear regression, and exploratory factor analysis (EFA) were the main statistical methods we used.

**Results:**

608 participants completed the questionnaires with a response rate of 97.9%. The mean age of the participants was 48.0 years (SD=9.6). About 33% were illiterate or only finished primary school education. Almost half participants (47.4%) only adopted chemotherapy. HRQOL was significantly different with regard to patients' social-demographic and clinical characteristics. Age, residence, educational level, employment status, and TNM stage were five significant predictors for global health status. Pain, dyspnea, sexual enjoyment, and systemic therapy side-effect were main subscales which had a significant impact on the global health status for patients in different TNM stage. The EFA result suggested that QLQ-C30 and QLQ-BR23 were complementary questionnaires.

**Conclusions:**

The EORTC QLQ-C30 and QLQ-BR23 questionnaires provide complementary information regarding breast cancer patients' HRQOL, and depending on the different cancer staging functional/symptom scales which significantly contributed to the overall HRQOL differed.

## 1. Introduction

Breast cancer is the second most common cancer in the world and by far the most frequent cancer among women with an estimated 1.67 million new cancer cases diagnosed in 2012 (25% of all cancers), and it ranks as the fifth cause of death from cancer overall (522,000 deaths) [1]. In 2013, breast cancer is the most prevalent malignant cancer and the fifth leading cause of cancer death in Chinese women [2, 3]. It has also been predicted by the International Agency for Research on Cancer that in 2020 the incidence and mortality of breast cancer in China would be 215,800 and 57,500, respectively [4].

Patient reported outcomes measures (PROMs), especially health-related quality of life (HRQOL) instruments, have increasingly been collected in randomised controlled trials (RCTs) to inform patient-centered care, clinical decision-making, and health policy or reimbursement decisions [5, 6]. Compared with the traditional assessment of clinical outcomes, HRQOL information plays an important role in breast cancer research [7]. Being diagnosed and living with breast cancer are a very stressful experience that may negatively affect multiple aspects of an individual's HRQOL and can have a long-term effect on HRQOL after treatment [8–10].

Along with the increasing number of breast cancer patients and the longer survival due to early detection programs and advancement in medical technology, accurately assessing HRQOL of breast cancer patients is crucial [11, 12]. By far, one of the most popular cancer-specific HRQOL instruments is the European Organization for Research and Treatment (EORTC) Quality of Life Questionnaire-C30 (QLQ-C30) [13]. The QLQ-C30 also has additional modules which can be used to address aspects which are of particular importance to patients with specific cancers. The breast cancer-specific module QLQ-BR23, a supplement to the general cancer questionnaire QLQ-C30, was developed to identify unique concerns to breast cancer patients [14].

Internationally, there are several studies assessing the HRQOL among breast cancer patients using the EORTC QLQ-C30 and QLQ-BR23 [7, 15–17]. The simplified Chinese versions of the above two questionnaires, which are commonly referred to as EORTC QLQ-BR53, have been validated in mainland China, demonstrating the validity, reliability and responsiveness in breast cancer patients [18, 19]. Although the QLQ-BR23 was developed to be used as an extension of the QLQ-C30; the literature is mixed as to whether to use both or either one of them in breast cancer patients [20].

The aims of this study are twofold: firstly, to explore which dimensions of HRQOL play more important roles in breast cancer patients' overall quality of life in China and, secondly, to explore the latent factor structure and the potential complementary relationship between these two EORTC questionnaires in breast cancer patients.

## 2. Patients and Methods

### 2.1. Patients

Participants were recruited from women breast cancer patients who have been treated in Qingdao Municipal Hospital, China, between October 2014 and February 2015. A total of 621 female breast cancer patients who underwent inpatient treatment from the oncology wards were interviewed. Patients clearly diagnosed with breast cancer and more than 18 years old were selected as an inclusion criterion. The exclusion criterions were as follows: (1) being unwilling to give an informed consent or (2) being unable to understand the questionnaires, or (3) being combined with other serious chronic diseases, such as cardiovascular or cerebrovascular diseases, psychosis, or (4) being younger than 18 years old at the time of the survey. Informed consent was obtained from all participants after a detailed explanation of the study. Ethical approval was obtained from the Ethics Review Board of the School of Public Health, Shandong University (Reference No. 20131002), and the research adhered to the tenets of the Declaration of Helsinki.

### 2.2. Tools and Measures

The participants were interviewed face to face one day prior to discharge. During the interview, information on sociodemographic characteristics of the participants was obtained, while the clinical information was collected by the interviewer based on the medical records. The HRQOL was self-assessed by each patient using the simplified Chinese version of the EORTC QLQ-BR53 questionnaires.

The EORTC QLQ-C30 is a reliable and valid questionnaire developed to assess the quality of life (QOL) of cancer patients, which has been translated and validated in over 100 languages and is used in more than 3,000 studies worldwide [21]. QLQ-C30 consists of 30 items (coded Q1-Q30), including a global health status (GHS)/QOL scale, five multi-item functional subscales (physical /role /emotional /cognitive /social functioning), and several single/multi-item symptomatic subscales (fatigue /nausea and vomiting /pain /dyspnoea /insomnia /appetite loss /constipation /diarrhea /financial difficulties) [13]. The EORTC QLQ-BR23 (coded BR1-BR23), a 23-item breast cancer-specific supplemental module, is meant for use among patients varying in disease stage and treatment modality. The module incorporates five multi-item scales to assess systemic therapy side-effects, arm symptoms, breast symptoms, body image, and sexual functioning. In addition, three single items assess sexual enjoyment, hair loss, and future perspective [14].

The QLQ-BR53 is rated on a four-level Likert scales response system, from 1 “not at all” to 4 “very much.” Except for the GHS items, Q29 and Q30, a seven-level Likert scale is used, from 1 “very poor” to 7 “excellent.” The time frame of the questionnaires is “during the past week,” except for the sexual functioning and sexual enjoyment (“during the past four weeks”). Scales raw scores are calculated by averaging items within scales. The raw score of the participants' responses are then linearly transformed to a 0–100 score according to the official EORTC scoring manual [22]. The score ranges from 0 to 100, with a higher score indicating a better quality of life for the functioning and GHS but a poorer quality of life for severe symptomatic problems. The GHS scale was used as the overall summary measure, where a high score represents a high overall quality of life. The level of self-assessed GHS helps in predicting survival, which is especially important among survivors to improve the QOL.

For all scales, the raw score, RS, is the mean of the component items:

(1) $$\begin{array}{c} \text{Raw Score}=\text{RS}=\frac{\left(I_{1}+I_{2}+\cdots +I_{n}\right)}{n}. \end{array}$$

Standard Score, SS, for global health status/functional scales are calculated as

(2) $$\begin{array}{c} \text{SS}=\left\{\;1-\frac{\left(\text{RS}-1\right)}{\text{range}}\;\right\}\times 100. \end{array}$$

And SS for symptom scales/items are calculated as

(3) $$\begin{array}{c} \text{SS}=\left\{\;\frac{\left(\text{RS}-1\right)}{\text{range}}\;\right\}\times 100. \end{array}$$

### 2.3. Statistical Analysis

Descriptive statistics was used to summarize sociodemographic and clinical characteristics of the participants. Normality tests were carried out for QOL scores. As all QOL scores were non-normally distributed, nonparametric tests (i.e., Mann–Whitney U test and Kruskal-Wallis test) were performed to determine whether differences in the mean score of QOL across sociodemographic and clinical parameters of the participants were significant. Differences were considered significant if p value was equal to or less than 0.05.

Regarding aim 1, step-wise multiple linear regression was conducted to investigate the statistically significant predictors associated with global QOL. Regarding aim 2, the Spearman rank correlation coefficients between QLQ-C30 and QLQ-BR23 subscales were firstly calculated, with correlation coefficient > 0.5 indicating a moderate association and a value < 0.5 representing a weak association [23]. Next, the latent factor structure was explored by using an exploratory factor analysis (EFA). The number of factors was extracted according to the eigenvalue (i.e., eigenvalue >1). To account for potential correlations among factors, rotation was performed using an oblique Promax method. Data were analyzed using a SPSS software package (SPSS version 22.0 Inc., Chicago, IL, USA).

## 3. Results

### 3.1. Sociodemographic and Clinical Characteristics

Six hundred and twenty-one women patients with confirmed diagnosis of breast cancer were interviewed. Of the total 621 patients, 13 were deleted due to incomplete answers. Finally, a valid sample of 608 patients (97.9%) was analyzed. The full sample sociodemographic and clinical characteristics were presented in Table 1.

### 3.2. QOL Scores by QLQ-C30 and QLQ-BR23

Table 2 showed the detailed QOL scores measured by QLQ-C30 and QLQ-BR23. The mean ± standard deviation (SD) score of GHS was 53.8±14.7. Among functional scales, more severe impairments were observed among future perspective (51.5±31.4), body image (64.9±25.0), and social functioning (69.9±24.6). Regarding symptom scales, more severe impairments were observed on insomnia (31.4±24.4), fatigue (34.0±18.1), financial difficulties (34.6±28.7), and upset by hair loss (38.6±30.3).

### 3.3. QOL Scores by Characteristics of the Participants and the Predictors of Global Health Status (GHS)/QOL

Table 3 presented main differences of QOL scores across clinical characteristics (while more detailed data about QOL by sociodemographic and clinical characteristics can be found in the supplementary materials (available here)). There were no significant differences for both QLQ-C30 and QLQ-BR23 functional scales among patients in different TNM stage. While significant differences were observed in QLQ-C30 symptom scales (including fatigue, nausea and vomiting, pain, dyspnea, appetite loss, and constipation). Patients who received chemotherapy only had lower scores in GHS as well as symptom scales while higher scores in functional scales than those that adopted other types of treatment.

Table 4 showed that among patient characteristics (Panel A), age, residence, educational level, employment status, and TNM stage were five statistically significant predictors for GHS. However, when further investigating the factors by the TNM stages, it can be seen that, in early stages (i.e., TNM stages 0-II), age was insignificant and marital status was significant, while, in advanced stages (i.e., TNM stages III-IV), marital status and employment status were insignificant.

The significant functional/symptom subscales which impacted on the global health status were reported in Panels B and C in Table 4. According to the different stages of cancer progressing, the significant scales in QLQ-C30 differed. In early stages, pain, dyspnea, and fatigue were significant, while, in advanced stages, role functioning, cognitive functioning, emotional functioning, nausea and vomiting, pain, and dyspnea were all significantly impacted on the global health status. Among the QLQ-BR23 scales, the same pattern of significant predictors was identified between different stages of breast cancer patients.

### 3.4. Spearman's Rank Correlation and EFA Factor Structure of QLQ-C30 and QLQ-BR23

Table 5 showed Spearman's rank correlation coefficients between QLQ-C30 and QLQ-BR23 scales. Generally speaking the correlations were weak, with the highest correlation coefficient of 0.428 (between “financial difficulties” in QLQ-C30 and “systemic therapy side-effects” in QLQ-BR23).

The exploratory factor analysis result was reported in Table 6. A total of 10 factors, which explained 61% of the variance, were extracted based on the eigenvalues (eigenvalues>1; Kaiser-Meyer-Olkin test=0.939; all communalities >0.34). As can be seen, QLQ-C30 and QLQ-BR23 items are attached to different latent factors, with the only exception observed in the third and fourth factor.

## 4. Discussion

By conducting a cross-sectional survey with 608 breast cancer patients, this study investigated two important issues that have not been explored in mainland China: what the important functional/symptom scales as well as patient characteristics that contributed to the overall quality of life of breast cancer patients are and whether those scales differ by different stages of cancer progressing and, furthermore, whether each of the QLQ-C30 and QLQ-BR23 provides unique information for the breast cancer patients and thus serves as a complementary measure to each other.

The breast cancer patients in our study had a mean GHS score of 53.8 (SD=14.7), lower than the EORTC QLQ-C30 Reference Values (mean=61.8, SD=24.6) [24], which might be explained by the larger proportion of patients receiving chemotherapy only as the type of treatment in our study (47.4%). It was reported that patients receiving chemotherapy might experience several side-effects that negatively affected their QOL [25]. This was confirmed in our study that patients received chemotherapy reported worse GHS comparing with those undergoing other types of treatment.

In our study, younger patients with a lower educational level reported significantly lower scores in sexual functioning and sexual enjoyment. In addition, the scores of sexual functioning and sexual enjoyment were pretty high comparing with similar researches [11, 26]. The findings were not consistent with the previous ones that disrupted sexual functioning or unsatisfactory sexual life was related to poorer HRQOL at younger age, treatment with chemotherapy, and emotional distress [27–29]. There might be an overestimation and the main potential reason may be the unique culture background. Sexuality is considered a private topic and is not openly discussed in public in China [30]. Thus, Chinese women are more conservative about the sex-related topics; the older or the lower-educated patients are more likely to avoid answering these questions or choose a random answer. As a disease closely related to women's identities, breast cancer affected the perception of sexuality and their own personal image [29]. Therefore, more attention should be paid to Chinese women with breast cancer on sexual problems.

Age, residence, educational level, employment status, and TNM stage were five significant predictors for overall quality of life according to the result of multiple linear regression. According to Wong-Kim et al., after being diagnosed with breast cancer, the Chinese women were more concerned about survival and may put a greater emphasis on recuperating from their illness rather than worrying about body image and their marital relationship. But, for traditional Chinese women, they did not want to burden their families with their deteriorating health [31]. What is more, patients with early breast cancer may be concerned about premature menopause leading to loss of fertility, recurrence, body image, and sexuality [32, 33], which could have a negative effect on their overall quality of life. Generally, better GHS scores were found in patients with stable job security, who were more likely to seek access to information and resources for problem solving. And they could be more compliant with the therapeutic regime because they had little problem in financial difficulties and felt more secure. However, when further investigating the factors by TNM stages, age was insignificant for early stage patients (TNM stages 0–II), and marital and employment status were insignificant for patients in advanced stages (TNM stages III-IV). The certain reasons were not clear at present, which remains to be discussed. Qualitative research can be considered to be applied to investigate the potential reasons in the future.

According to the different stages of cancer progressing, the significant subscales differed. For all patients, an increased level of systemic therapy side-effect was linked to a decrease on GHS score. Increased levels of pain and fatigue were associated with a 0.107 and 0.212 GHS score decreased for early stage patients, and increased levels of role and cognitive functioning were associated with a 0.119 and 0.211 GHS score increased for advanced stage patients. These findings were consistent with former studies that treatment side-effects (even those of lower grade) may induce symptoms such as nausea and vomiting, pain, and fatigue, which are not life-threatening but can adversely impact patients' well-being and are associated with worse HRQOL [34]. While an increased level of sexual enjoyment associated with a decrease on GHS score and the aggravation of dyspnea associated with an increase on GHS score were inconsistent with our common sense. The subjective perception of HRQOL might not necessarily be in accordance with relevant clinical factors [35]. Patients' expectation might change with time and psychological response to breast cancer might be independent of cancer stage [36]. If a person is confronting an incurable condition their values will shift and patients certainly assign different values to hypothetical health states than outsiders [20]. Therefore, more attention should be paid to improve patients' HRQOL through dealing with the particular impaired function or symptom for it has been quite difficult to extend lifespan for the advanced breast cancers or the long-term survivors.

This study also investigated the complementary roles between two EORTC questionnaires in breast cancer. The correlation and factor analysis have been used to confirm the construct and criterion-related validity of the simplified Chinese version of the QLQ-BR53; however, the factor analyses were applied separately to QLQ-C30 and QLQ-BR23 [18, 19]. In our study, Spearman's rank correlation and an EFA analysis were used to assess the degree of conceptual overlap between QLQ-C30 and QLQ-BR23. The results showed that there were only weak correlations between the two QOL instruments of EORTC QLQ-C30 and QLQ-BR23. The EFA results showed cumulative contribution rates > 61%, indicating a good extraction effect. Ten common factors extracted were not completely conformed to the theory structure of the EOTRC QLQ-BR53 scales, but the EFA results mostly confirmed the original scoring algorithm. Among 10 latent factors, only factors 3 and 4 were composed of both QLQ-C30 items and one QLQ-BR23 item, further confirming a complementary role between these two questionnaires.

This study has two main limitations. Firstly, the patients were recruited from eastern China and the conclusions may not be applicable to the whole Chinese populations. Second, this was a cross-sectional study. It will be important to further assess the changes on HRQOL using a longitudinal survey design.

In conclusion, clinical characteristics are relevant factors influencing the QOL of women breast cancer patients in eastern China. Age, residence, educational level, employment status, and TNM stage were five significant predictors for GHS. Pain, dyspnea, sexual enjoyment, and systemic therapy side-effect were main subscales which had a significant impact on the global health status for all patients. As complementary questionnaires, it is recommended that the EORTC QLQ-C30 and QLQ-BR23 should be applied to assess HRQOL at the same time. The results of this study enriched our understanding of using EORTC QLQ-C30 and QLQ-BR23 questionnaires in China.

## Figures and Tables

**Table 1:** Sociodemographic and clinical characteristics of the participants (n=608).

**Characteristics**	**Number**	**Percentage (**%**)**
**Age (Mean=48.0, SD=9.6)**		
<45	220	36.2
45-54	224	36.8
≥55	164	27.0
**Residence(house locality)**		
Rural	299	49.2
Urban	309	50.8
**Marital status**		
Single	27	4.4
Married	539	88.7
Divorced/Widow	42	6.9
**Employment status**		
Urban employee	396	65.1
Peasants	156	25.7
Unemployment	47	7.7
Others	9	1.5
**Educational level**		
Illiteracy or Primary school	200	32.9
Secondary school	128	21.1
High school/technical secondary school	131	21.5
University degree and above	149	24.5
**Annual household income (Chinese Yuan, CNY)**		
<30,000	161	26.5
30,000-80,000	262	43.1
80,001-150,000	153	25.2
>150,000	32	5.2
**Metastatic breast cancer state (State M)**		
Yes	141	23.2
No	467	76.8
**TNM Stage**		
0-I	175	28.8
II	142	23.4
III	218	35.8
IV	73	12.0
**Disease duration, months (Mean=38.2, SD=40.9)**		
≤12	190	31.2
13-36	197	32.4
37-60	112	18.4
≥61	109	18.0
**Menopausal Status**		
Pre-menopausal	274	45.1
Post-menopausal	334	54.9
**Type of treatment**		
Chemotherapy only	288	47.4
Others	320	52.6

**Note:**

TNM: tumor, node, and metastases classification of malignant tumors.

Exchange rate: according to the Organization for Economic Cooperation and Development (OECD) data (https://data.oecd.org/conversion/exchange-

rates.htm), the average annual exchange rate between US$ and CNY in 2014 was US$1 = CNY 6.143, and in 2015 it was US$1 = CNY 6.227.

**Table 2:** Quality of life scores for all participants (n=608).

**EORTC Item**	**Mean score**	**SD**	**Median**
**Panel A: QLQ-C30 Questionnaire**			

**Global Health Status**	53.8	14.7	50.0
**Functional Scales**			
Physical functioning	75.5	17.2	80.0
Role functioning	77.4	25.5	83.3
Emotional functioning	74.2	19.7	75.0
Cognitive functioning	76.9	19.5	83.3
Social functioning	69.9	24.6	66.7
**Symptom Scales**			
Fatigue	34.0	18.1	33.3
Nausea and vomiting	19.0	21.5	16.7
Diarrhoea	10.4	18.9	0.0
Financial difficulties	34.6	28.7	33.3
Pain	28.9	19.9	33.3
Dyspnoea	17.2	22.2	0.0
Insomnia	31.4	24.4	33.3
Appetite loss	24.1	25.3	33.3
Constipation	24.6	26.4	33.3

**Panel B: QLQ-BR23 Questionnaire**			

**Functional Scales**			
Body image	64.9	25.0	66.7
Sexual functioning	89.0	15.9	100.0
Future perspective	51.5	31.4	66.7
Sexual enjoyment	88.3	19.6	100.0
**Symptom Scales**			
Systemic therapy side effects	24.7	16.9	23.8
Breast symptoms	17.1	19.8	8.3
Arm symptoms	20.2	19.6	16.7
Upset by hair loss	38.6	30.3	33.3

SD, standard deviation

**Table 3:** Quality of life scores (mean ± SD) by TNM stage, type of treatment.

**EORTC Items**	**TNM stage**				**P**	**Type of treatment**		**P**
	**0-I**	**II**	**III**	**IV**		**Chemotherapy only**	**Others**	
**Panel A: QLQ-C30 Questionnaire**								

GHS	55.1±13.3	54.0±15.8	52.6±15.1	53.9±14.0	0.497	51.8±12.8	55.6±15.9	<0.001^*∗*^
**Functional scales**								
PF	75.5±17.2	78.5±15.1	74.4±18.0	73.0±18.4	0.068	78.5±16.2	72.8±17.7	<0.001^*∗*^
RF	76.9±25.0	81.1±25.2	76.2±25.4	74.4±27.5	0.112	81.0±23.3	74.1±27.0	0.001^*∗*^
EF	75.0±18.3	77.0±19.5	72.3±20.6	72.7±20.3	0.170	75.9±18.7	72.7±20.5	0.052
CF	78.2±18.1	79.2±17.4	74.8±21.8	75.3±18.7	0.303	79.0±18.3	74.9±20.3	0.012^*∗*^
SF	71.5±25.8	71.8±22.8	68.6±25.3	66.2±22.6	0.180	71.5±23.5	68.5±25.5	0.118
**Symptom scales**								
FA	34.4±16.4	30.9±18.3	35.0±18.5	36.2±19.8	0.017^*∗*^	32.0±16.1	35.8±19.5	0.016^*∗*^
NV	19.5±21.2	14.0±18.6	20.9±23.2	21.7±20.7	0.010^*∗*^	15.0±19.1	22.6±22.8	<0.001^*∗*^
DI	10.5±18.2	7.5±16.1	12.2±21.5	10.5±16.5	0.211	7.5±16.0	13.0±20.8	0.001^*∗*^
FI	33.9±31.0	33.6±28.0	35.0±27.6	37.4±27.7	0.568	33.6±26.8	35.6±30.2	0.545
PA	29.4±18.8	25.0±19.5	30.3±20.6	30.8±20.0	0.025^*∗*^	26.1±18.7	31.3±20.5	0.001^*∗*^
DY	17.9±21.7	12.2±18.0	19.1±23.7	19.6±25.4	0.039^*∗*^	12.7±19.3	21.2±23.9	<0.001^*∗*^
SL	32.2±24.2	26.8±22.5	32.7±24.6	34.7±26.9	0.092	30.2±24.1	32.5±24.6	0.225
AP	23.6±23.4	18.3±23.0	26.6±26.7	28.8±27.9	0.009^*∗*^	21.5±23.4	26.3±26.7	0.038^*∗*^
CO	26.5±26.6	19.0±23.3	26.6±28.0	25.1±25.3	0.048^*∗*^	23.0±26.1	26.0±26.6	0.136

**Panel B: QLQ-BR23 Questionnaire**								

**Functional scales**								
BRBI	65.2±24.3	67.6±26.4	62.9±24.8	64.8±24.4	0.355	65.9±25.9	64.0±24.2	0.090
BRSEF	87.1±16.5	91.1±15.7	89.2±15.7	88.6±15.2	0.081	92.4±14.0	85.9±16.9	<0.001^*∗*^
BRFU	51.0±31.5	53.5±33.9	49.7±30.9	53.9±27.6	0.600	51.4±30.8	51.6±32.0	0.902
BRSEE	87.5±18.1	91.2±17.8	87.6±21.6	87.2±19.7	0.153	92.8±14.6	84.3±22.4	<0.001^*∗*^
**Symptom scales**								
BRST	24.8±16.2	21.8±15.7	26.5±17.8	24.8±17.1	0.097	23.0±15.6	26.3±17.8	0.023^*∗*^
BRBS	17.1±18.6	13.2±16.9	18.6±21.5	20.3±21.8	0.075	14.6±19.5	19.4±19.8	<0.001^*∗*^
BRAS	20.4±18.4	17.2±18.5	20.7±20.8	24.2±20.6	0.083	17.8±19.6	22.4±19.4	0.001^*∗*^
BRHL	38.6±30.5	37.8±32.1	40.3±29.9	34.7±27.3	0.562	39.7±30.3	37.6±30.3	0.450

SD, standard deviation.

GHS, global health status; PF, physical functioning; RF, role functioning; EF, emotional functioning; CF, cognitive functioning.

SF, social functioning; FA, fatigue; NV, nausea and vomiting; DI, diarrhoea; FI, financial difficulties; PA, pain; DY, dyspnoea.

SL, insomnia; AP, appetite loss; CO, constipation; BRBI, body image; BRSEF, sexual functioning; BRFU, future perspective.

BRSEE, sexual enjoyment; BRST, systemic therapy side effect; BRBS, breast symptoms; BRAS, arm symptoms; BRHL, upset by hair loss.

**Table 4:** Final model of predictors for Global Health Status scores.

	**GHS of all participants**	**GHS of patients in TNM Stage 0–II**	**GHS of patients in TNM Stage III-IV**
	**Coefficient (SE)**	**Coefficient (SE)**	**Coefficient (SE)**

**Panel A: Patient characteristics**			

**(Constant)**	87.720(5.150)^*∗∗*^	102.608(6.136)^*∗∗*^	64.272(7.553)^*∗∗*^
**Age**	-0.146(0.053)^*∗*^	-0.039(-0.774)	-0.183(0.071)^*∗*^
**Residence (ref: urban)**			
rural	5.454(1.220)^*∗∗*^	5.848(1.834)^*∗*^	2.920(1.365)^*∗*^
**Marital status (ref: single)**			
Married	-0.047(-1.295)	-15.586(4.604)^*∗*^	-0.001(-0.015)
Divorced/widow	-0.023(-0.660)	-14.775(5.079)^*∗*^	-0.025(-0.506)
**Educational level (ref: illiteracy /primary school)**			
Secondary school	-0.020(-0.582)	-0.034(-0.673)	0.024(0.509)
High school/technical secondary school	0.043(1.098)	0.085(1.461)	0.046(0.906)
university degree and above	6.432(1.275)^*∗∗*^	5.418(1.920)^*∗*^	8.956(1.699)^*∗∗*^
**Employment status (ref: urban employee)**			
peasants	-4.947(1.395)^*∗∗*^	-4.515(1.898)^*∗*^	-0.099(-1.877)
unemployment	-4.336(1.984)^*∗*^	-0.092(-1.791)	-0.029(-0.647)
others	-0.037(-1.134)	-0.019(-0.395)	-0.010(-0.222)
**TNM Stage (ref: 0-II)**			
III-IV	-1.912(0.952)^*∗*^	**——**	**——**

**Panel B: QLQ-C30 scales**			

Role functioning	0.081(0.028)^*∗*^	0.050(0.711)	0.119(0.039)^*∗*^
Cognitive functioning	0.077(1.520)	0.035(0.510)	0.211(0.058)^*∗∗*^
Emotional functioning	-0.066(-1.392)	-0.047(-0.720)	-0.131(0.056)^*∗*^
Nausea and vomiting	0.046(1.017)	-0.056(-0.874)	0.099(0.042)^*∗*^
Pain	-0.125(0.038)^*∗*^	-0.107(0.050)^*∗*^	-0.140(0.055)^*∗*^
Dyspnoea	0.128 (0.027)^*∗∗*^	0.123(0.042)^*∗*^	0.104(0.035)^*∗*^
Fatigue	-0.135(0.043)^*∗*^	-0.212(0.058)^*∗∗*^	-0.130(-1.771)
insomnia	-0.049(0.023)^*∗*^	-0.037(-0.670)	-0.082(-1.507)

**Panel C: QLQ-BR23 scales**			

Sexual enjoyment	-0.230(0.027)^*∗∗*^	-0.273(0.041)^*∗∗*^	-0.194(0.035)^*∗∗*^
systemic therapy side effect	-0.213(0.042)^*∗∗*^	-0.144(0.050)^*∗*^	-0.185(0.055)^*∗*^
Arm symptoms	0.078(0.034)^*∗*^	0.107(1.711)	0.120(1.912)
Body image	-0.053(0.025)^*∗*^	-0.075(-1.223)	-0.092(-1.609)

**Note: **stepwise regression was applied in the multiple linear regression analysis; a constant was included in each model.

SE, Standard Error; *∗∗* and *∗* indicate P < 0.01 and P < 0.05, respectively.

**Table 5:** Spearman's rank correlation coefficients among QLQ-C30 and QLQ-BR23 scales.

**QLQ-C30**																	
			**GHS**	**Functional scales**					**Symptom scales**								
				**PF**	**RF**	**EF**	**CF**	**SF**	**FA**	**NV**	**DI**	**FI**	**PA**	**DY**	**SL**	**AP**	**CO**

**QLQ-BR23**	**Functional scales**	**BRBI**	0.021	0.149	0.088	0.111	0.129	0.011	-0.083	-0.060	-0.066	-0.376	-0.019	-0.103	-0.033	-0.066	-0.012
		**BRSEF**	-0.349	0.192	0.206	0.172	0.186	0.154	-0.159	-0.210	-0.087	-0.212	-0.176	-0.149	-0.124	-0.193	-0.067
		**BRFU**	0.056	0.155	0.040	0.097	0.094	0.014	-0.065	-0.075	-0.109	-0.371	-0.017	-0.125	-0.012	-0.041	-0.058
		**BRSEE**	-0.383	0.224	0.247	0.194	0.217	0.181	-0.188	-0.188	-0.073	-0.215	-0.222	-0.178	-0.125	-0.201	-0.079
	**Symptom scales**	**BRST**	-0.138	-0.155	-0.103	-0.106	-0.134	-0.034	0.080	0.106	0.063	0.428	0.056	0.092	0.022	0.065	0.023
		**BRBS**	0.058	-0.250	-0.237	-0.204	-0.198	-0.121	0.195	0.145	0.049	0.350	0.170	0.177	0.066	0.179	0.029
		**BRAS**	0.019	-0.235	-0.200	-0.187	-0.170	-0.131	0.158	0.116	0.090	0.412	0.097	0.129	0.025	0.094	0.010
		**BRHL**	-0.215	-0.098	-0.018	-0.040	-0.059	0.016	0.036	0.014	0.041	0.289	-0.023	0.057	0.013	0.001	0.022

**Note: **p<0.01.

GHS, global health status; PF, physical functioning; RF, role functioning; EF, emotional functioning; CF, cognitive functioning.

SF, social functioning; FA, fatigue; NV, nausea and vomiting; DI, diarrhoea; FI, financial difficulties; PA, pain; DY, dyspnoea.

SL, insomnia; AP, appetite loss; CO, constipation; BRBI, body image; BRSEF, sexual functioning; BRFU, future perspective.

BRSEE, sexual enjoyment; BRST, systemic therapy side effect; BRBS, breast symptoms; BRAS, arm symptoms; BRHL, upset by hair loss.

**Table 6:** Exploratory factor analysis (pattern matrix).

	**Factor Loadings**									
	**Factor 1**	**Factor 2**	**Factor 3**	**Factor 4**	**Factor 5**	**Factor 6**	**Factor 7**	**Factor 8**	**Factor 9**	**Factor 10**
**BR12**	0.952									
**BR11**	0.942									
**BR10**	0.870									
**BR9**	0.769									
**BR13**	0.637									
**BR8**	0.352		0.349							
**BR5**										
**BR23**		0.921								
**BR21**		0.907								
**BR20**		0.897								
**BR22**		0.865								
**BR18**		0.598								0.365
**BR19**		0.591								
**BR17**		0.525		0.311						
**Q14**			0.854							
**Q15**			0.770							
**Q13**			0.668							
**Q16**			0.641							
**Q17**			0.478							
**BR7**			0.404							
**Q11**			0.403							
**Q12**			0.382	0.327						
**Q6**				0.930						
**Q7**				0.896						
**Q5**			0.327	0.490						
**Q2**				0.470						
**Q3**			0.336	0.458						
**Q8**			0.364	0.452						
**Q10**				0.429						
**Q4**				0.397						
**Q1**				0.382						
**Q9**				0.334						
**Q27**					0.910					
**Q28**					0.845					
**Q26**					0.817					
**Q24**					0.477					
**Q25**					0.440					
**Q21**						0.893				
**Q22**						0.857				
**Q23**						0.777				
**Q20**						0.534				
**Q19**						0.398				
**Q18**						0.324				
**BR15**							0.900			
**BR14**							0.863			
**BR16**							0.382			
**Q29**								-0.918		
**Q30**								-0.859		
**BR1**									0.775	
**BR2**									0.767	
**BR3**									0.637	
**BR4**										0.537
**BR6**										0.508

***Note*. **extraction method: maximum likelihood. Rotation method: Promax with Kaiser normalization.

Loadings below 0.30 were not shown.

## Data Availability

The data used to support the findings of this study are available from the corresponding author upon request.

## Supplementary Materials

Supplementary Materials More detailed data about quality of life scores by sociodemographic and clinical characteristics of the participants were displayed in the supplementary materials. Differences in the mean score of quality of life across sociodemographic and clinical parameters of the participants were considered significant if p value was equal to or less than 0.05. Table S1: quality of life scores by age, residence (house locality), and marital status; Table S2: quality of life scores by educational level and medical security; Table S3: quality of life scores by annual household income and disease duration; Table S4: quality of life scores by metastatic breast cancer state (State M) and menopausal status.

## References

[1] Ferlay J., Soerjomataram I., Dikshit R. (2014). Cancer incidence and mortality worldwide: sources, methods and major patterns in GLOBOCAN 2012. *International Journal of Cancer*.

[2] Zuo T. T. (2017). Female breast cancer incidence and mortality in China, 2013. *Thoracic Cancer*.

[3] Chen W., Zheng R., Baade P. D. (2016). Cancer statistics in China, 2015. *CA: A Cancer Journal for Clinicians*.

[4] IARC GLOBOCAN 2012: Estimated Cancer Incidence, Mortality and Prevalence Worldwide in 2012--Online Analysis/Prediction. http://globocan.iarc.fr/Pages/burden_sel.aspx.

[5] Lipscomb J., Reeve B. B., Clauser S. B. (2007). Patient-reported outcomes assessment in cancer trials: taking stock, moving forward. *Journal of Clinical Oncology*.

[6] Calvert M., Blazeby J., Altman D. G., Revicki D. A., Moher D., Brundage M. D. (2013). Reporting of patient-reported outcomes in randomized trials: the CONSORT PRO extension. *Journal of the American Medical Association*.

[7] Montazeri A. (2008). Health-related quality of life in breast cancer patients: a bibliographic review of the literature from 1974 to 2007. *Journal of Experimental & Clinical Cancer Research*.

[8] Ganz P. A., Coscarelli A., Fred C., Kahn B., Polinsky M. L., Petersen L. (1996). Breast cancer survivors: psychosocial concerns and quality of life. *Breast Cancer Research & Treatment*.

[9] Fallowfield L. (2002). Quality of life: a new perspective for cancer patients. *Nature Reviews Cancer*.

[10] Cui Y., Shu X. O., Gao Y. (2004). The long-term impact of medical and socio-demographic factors on the quality of life of breast cancer survivors among Chinese women. *Breast Cancer Research and Treatment*.

[11] Yusuf A., Ab Hadi I. S., Mahamood Z., Ahmad Z., Keng S. L. (2013). Quality of life in Malay and Chinese women newly diagnosed with breast cancer in Kelantan, Malaysia. *Asian Pacific Journal of Cancer Prevention*.

[12] Perry S., Kowalski T. L., Chang C.-H. (2007). Quality of life assessment in women with breast cancer: benefits, acceptability and utilization. *Health and Quality of Life Outcomes*.

[13] Aaronson N. K., Ahinedzai S., Bergman B. (1993). The European Organization for Research and Treatment of Cancer QLQ-C30: a quality-of-life instrument for use in international clinical trials in oncology. *Journal of the National Cancer Institute*.

[14] Sprangers M. A., Groenvold M., Arraras J. I. (1996). The European Organization for Research and Treatment of Cancer breast cancer-specific quality-of-life questionnaire module: first results from a three-country field study. *Journal of Clinical Oncology*.

[15] Ho P. J., Gernaat S. A., Hartman M., Verkooijen H. M. (2018). Health-related quality of life in Asian patients with breast cancer: a systematic review. *BMJ Open*.

[16] Rahou B. H., El Rhazi K., Ouasmani F. (2016). Quality of life in Arab women with breast cancer: a review of the literature. *Health and Quality of Life Outcomes*.

[17] Kawatkar A., Gwadry-Sridhar F. (2006). PCN43 a systematic review of the eortc QLQ-BR23: descriptive health related quality of life instrument used in breast cancer. *Value in Health*.

[18] Wan C., Tang X., Tu X. M. (2007). Psychometric properties of the simplified Chinese version of the EORTC QLQ-BR53 for measuring quality of life for breast cancer patients. *Breast Cancer Research and Treatment*.

[19] Zhang Z., Zhang X., Wei L. (2017). Questionnaire to assess quality of life in patients with breast cancer—validation of the Chinese version of the EORTC QLQ-BR 53. *The Breast*.

[20] Reed E., Kössler I., Hawthorn J. (2012). Quality of life assessments in advanced breast cancer: Should there be more consistency?. *European Journal of Cancer Care*.

[21] EORTC QLQ-C30. http://groups.eortc.be/qol/eortc-qlq-c30.

[22] Fayers P. M., Aaronson N. K., Bjordal K. The EORTC QLQ-C30 scoring manual, 2001.

[23] Chang Y. (2016). Reliability and validity of the Chinese mandarin version of PedsQL 3.0 transplant module. *Health Qual Life Outcomes*.

[24] Scott N. W., Fayers P. M., Aaronson N. K. EORTC QLQ-C30 Reference Values, 2008.

[25] Montazeri A., Vahdaninia M., Harirchi I., Ebrahimi M., Khaleghi F., Jarvandi S. (2008). Quality of life in patients with breast cancer before and after diagnosis: an eighteen months follow-up study. *BMC Cancer*.

[26] Shen F.-R., Liu M., Zhang X., Feng Y.-H., Zhou L.-S., Chen Y.-G. (2012). Health-related quality of life among breast cancer patients and its influencing factor in a Chinese population. *Asian Pacific Journal of Cancer Prevention*.

[27] Kim K. R., Chung H. C., Lee E., Kim S. J., Namkoong K. (2012). Body image, sexual function and depression in Korean patients with breast cancer: modification by 5-HTT polymorphism. *Supportive Care in Cancer*.

[28] Jabson J. M., Donatelle R. J., Bowen D. J. (2011). Relationship between sexual orientation and quality of life in female breast cancer survivors. *Journal of Women's Health*.

[29] Ganz P. A., Rowland J. H., Desmond K., Meyerowitz B. E., Wyatt G. E. (1998). Life after breast cancer: understanding women's health-related quality of life and sexual functioning. *Journal of Clinical Oncology*.

[30] Wang F., Chen F., Huo X. (2013). A neglected issue on sexual well-being following breast cancer diagnosis and treatment among Chinese women. *PLoS ONE*.

[31] Wong-Kim E., Sun A., Merighi J. R., Chow E. A. (2005). Understanding quality-of-life issues in Chinese women with breast cancer: a qualitative investigation. *Cancer control : journal of the Moffitt Cancer Center*.

[32] Sharma N., Purkayastha A. (2017). Factors affecting quality of life in breast cancer patients: a descriptive and cross-sectional study with review of literature. *Journal of Mid-life Health*.

[33] Baucom D. H., Porter L. S., Kirby J. S., Gremore T. M., Keefe F. J. (2005). Psychosocial issues confronting young women with breast cancer. *Breast Disease*.

[34] Cortes J., Hudgens S., Twelves C. (2015). Health-related quality of life in patients with locally advanced or metastatic breast cancer treated with eribulin mesylate or capecitabine in an open-label randomized phase 3 trial. *Breast Cancer Research and Treatment*.

[35] Huang C.-C., Lien H.-H., Tu S.-H. (2010). Quality of life in Taiwanese breast cancer survivors with breast-conserving therapy. *Journal of the Formosan Medical Association*.

[36] Witek-Janusek L., Gabram S., Mathews H. L. (2007). Psychologic stress, reduced NK cell activity, and cytokine dysregulation in women experiencing diagnostic breast biopsy. *Psychoneuroendocrinology*.

