# Supplementary material for: Health-Related Quality of Life among Women Breast Cancer Patients in Eastern China
Source: Biomed Res Int. 2018 Jul 3;2018:1452635. doi: 10.1155/2018/1452635 (PMC6051298; doi:10.1155/2018/1452635)
Supplement: Supplementary Materials — More detailed data about quality of life scores by sociodemographic and clinical characteristics of the participants were displayed in the supplementary materials. Differences in the mean score of quality of life across sociodemographic and clinical parameters of the participants were considered significant if p value was equal to or less than 0.05. Table S1: quality of life scores by age, residence (house locality), and marital status; Table S2: quality of life scores by educational level and medical security; Table S3: quality of life scores by annual household income and disease duration; Table S4: quality of life scores by metastatic breast cancer state (State M) and menopausal status. [file 1452635.f1.pdf]

Supplementary Materials

Quality of life scores by socio-demographic and clinical characteristics of the participants

Abbreviations:

SD, standard deviation;

GHS, global health status; PF, physical functioning; RF, role functioning; EF, emotional functioning; CF, cognitive functioning;

SF, social functioning; FA , fatigue; NV, nausea and vomiting; DI, diarrhoea; FI, financial difficulties; PA, pain; DY, dyspnoea;

SL, insomnia; AP, appetite loss; CO, constipation; BRBI, body image; BRSEF, sexual functioning; BRFU, future perspective;

BRSEE, sexual enjoyment; BRST, systemic therapy side effect; BRBS, breast symptoms; BRAS, arm symptoms; BRHL, upset by hair loss.

Table S1 Quality of life scores (Mean ± SD) by Age, Residence (house locality), Marital status

EORTC Items	Age			P	Residence (house locality)		P	Marital status			P
	≤45	45-54	≥55		Urban	Rural		Married	Divorced/ Widow	Single	
**Panel A - QLQ-C30 Questionnaire**											
GHS	58.0±14.5	53.5±14.0	48.5±14.1	＜0.001*	53.8±14.3	53.8±15.1	0.782	52.9±14.7	58.3±11.2	65.7±13.5	＜0.001*
**Functional scales**											
PF	75.0±15.6	74.5±17.9	77.4±18.3	0.052	77.4±16.4	73.5±17.8	0.002*	76.5±17.3	64.9±15.6	72.1±12.9	＜0.001*
RF	78.4±24.4	75.6±26.1	78.3±26.2	0.383	79.2±25.0	75.5±26.0	0.053	78.3±25.9	64.7±18.8	77.8±21.7	＜0.001*
EF	75.0±18.4	73.0±20.2	74.9±20.7	0.444	76.3±19.1	72.1±20.1	0.014*	75.6±19.7	60.7±18.5	68.8±13.2	＜0.001*
CF	77.9±18.1	75.1±19.2	77.9±21.5	0.096	79.0±18.9	74.7±19.8	0.004*	77.9±19.4	62.3±18.0	79.6±12.5	＜0.001*
SF	71.1±22.9	70.3±24.4	67.8±27.1	0.067	72.9±23.5	66.8±25.3	0.003*	70.6±24.8	57.1±19.9	74.7±22.8	＜0.001*
**Symptom scales**											
FA	33.4±16.5	35.6±18.4	32.6±19.5	0.166	33.4±18.1	34.63±18.0	0.340	33.2±18.2	42.3±16.9	36.2±12.9	＜0.001*
NV	19.2±21.4	20.6±21.2	16.6±21.8	0.065	16.9±20.7	21.13±22.1	0.014*	17.3±20.6	41.3±22.2	18.5±18.1	＜0.001*
DI	11.5±18.3	11.9±20.6	6.9±16.7	0.007*	9.1±18.5	11.82±19.2	0.028*	8.6±17.3	31.7±25.4	13.6±16.7	＜0.001*
FI	33.5±27.9	34.2±28.7	36.8±29.7	0.529	31.3±28.0	38.13±28.9	0.002*	33.9±28.4	45.2±29.3	32.1±31.3	0.038*
PA	25.5±18.9	31.3±18.6	30.0±22.1	0.005*	27.2±20.3	30.60±19.2	0.009*	28.5±20.2	35.7±16.7	24.7±15.6	0.006*

DY	18.9±22.3	18.6±24.0	13.0±19.0	0.024*	15.6±21.7	18.84±22.6	0.052	15.5±21.1	37.3±26.7	21.0±21.0	＜0.001*
SL	32.0±24.7	33.3±25.6	28.0±21.8	0.158	32.4±25.4	30.43±23.3	0.476	31.3±24.5	39.7±22.4	21.0±21.0	0.006*
AP	24.1±26.1	26.2±26.2	21.1±22.8	0.221	22.4±25.5	25.75±25.1	0.056	22.9±25.0	43.6±24.9	16.0±19.3	＜0.001*
CO	24.7±26.1	26.5±26.6	21.9±26.2	0.185	23.4±25.8	25.86±26.9	0.271	23.4±25.7	45.2±30.2	17.3±19.3	＜0.001*
**Panel B - QLQ-BR23 Questionnaire**											
**Functional scales**											
BRBI	66.4±23.1	65.8±21.5	61.8±31.0	0.917	65.8±25.5	64.0±24.4	0.223	65.5±25.7	55.4±18.9	67.6±13.5	0.001*
BRSEF	85.0±17.0	89.2±16.2	94.1±12.2	＜0.001*	88.5±16.3	89.5±15.5	0.482	90.2±15.2	74.4±17.5	86.4±16.0	＜0.001*
BRFU	51.5±30.1	53.1±29.9	49.2±34.9	0.546	54.1±30.9	48.7±31.7	0.039*	51.8±31.7	46.0±32.9	54.3±21.0	0.503
BRSEE	84.5±22.0	87.7±20.3	94.4±12.5	＜0.001*	87.8±20.7	88.8±18.4	0.808	89.9±18.1	66.7±26.1	88.9±18.5	＜0.001*
**Symptom scales**											
BRST	24.8±16.9	24.4±16.7	25.0±17.2	0.932	22.7±17.5	26.7±16.0	＜0.001*	23.9±16.8	36.3±13.3	22.2±15.7	＜0.001*
BRBS	17.6±19.1	17.8±20.5	15.4±19.8	0.359	15.2±19.1	19.1±20.3	0.015*	15.5±19.5	34.9±18.0	20.7±15.6	＜0.001*
BRAS	18.3±18.0	21.7±19.9	20.8±21.2	0.258	18.4±18.9	22.1±20.3	0.016*	19.5±19.8	33.1±15.6	15.2±13.5	＜0.001*
BRHL	33.8±27.9	37.9±30.0	45.7±32.4	0.002*	35.9±30.1	41.2±30.2	0.025*	38.3±31.0	44.7±25.4	34.6±21.6	0.175*

Table S2 Quality of life scores (Mean ± SD) by Educational level, Medical security

EORTC Items	Educational level				P	Medical security				P
	Illiteracy or primary school	Secondary school	High school/ Technical secondary school	University degree and above		New rural cooperative medical system(NCMS)	Urban workers insurance	Urban residents insurance	others	
**Panel A - QLQ-C30 Questionnaire**										
GHS	50.4±12.0	52.3±14.4	52.3±15.4	61.0±15.2	＜0.001*	53.7±15.1	52.8±13.7	60.9±12.9	57.0±20.1	0.001*
**Functional scales**										
PF	78.0±15.6	75.2±18.4	75.9±18.4	72.0±16.8	0.001*	74.7±16.6	77.6±17.0	71.5±14.9	65.1±23.7	＜0.001*
RF	79.5±24.5	78.0±25.7	77.5±26.1	73.8±26.2	0.158	77.5±25.5	78.5±25.5	71.3±23.3	70.5±28.8	0.137
EF	76.8±19.6	74.1±19.1	74.8±21.0	70.4±18.8	0.013*	72.9±20.1	76.4±19.2	72.7±18.8	65.1±19.9	0.012*
CF	78.5±19.4	76.7±18.0	78.2±20.9	73.7±19.4	0.047*	75.8±19.6	79.4±18.9	70.8±16.9	67.3±22.8	0.001*
SF	67.8±25.8	68.5±23.3	72.7±25.2	71.4±23.3	0.247	68.2±25.6	72.7±24.0	62.5±19.4	65.4±23.1	0.028*
**Symptom scales**										
FA	31.7±17.3	34.5±19.5	35.2±18.0	35.6±17.8	0.026*	34.4±17.6	33.2±17.5	35.1±21.5	37.6±24.1	0.464
NV	15.6±18.5	19.8±22.4	20.4±23.5	21.7±22.1	0.088	19.1±21.8	17.3±20.2	22.9±24.6	32.0±24.0	0.010*
DI	6.3±13.1	11.7±22.0	9.7±18.2	15.4±21.8	＜0.001*	10.1±18.4	8.0±16.5	22.9±24.3	24.4±24.1	＜0.001*

FI	38.2±28.6	35.4±27.3	32.1±30.0	31.5±28.4	0.063	36.6±29.2	31.2±26.9	44.8±33.4	39.7±31.3	0.038*
PA	28.2±19.6	27.7±20.4	29.5±19.7	30.2±19.9	0.675	29.5±19.1	27.3±19.8	29.7±20.2	37.8±25.6	0.083
DY	11.5±16.9	18.0±24.5	18.3±24.2	23.3±23.1	＜0.001*	18.0±22.1	14.7±21.5	26.0±26.4	25.6±21.7	0.003*
SL	29.0±22.5	34.6±23.4	31.3±25.1	32.0±26.8	0.256	29.1±23.6	32.3±24.7	35.4±26.7	39.7±25.0	0.108
AP	20.3±22.6	24.0±24.7	27.2±27.7	26.4±26.6	0.117	24.1±25.3	22.9±23.8	27.1±31.0	33.3±32.7	0.520
CO	23.5±25.2	25.8±27.8	22.6±25.9	26.8±27.0	0.541	23.6±26.4	23.7±25.2	32.3±32.2	35.9±28.2	0.071
**Panel B - QLQ-BR23 Questionnaire**										
**Functional scales**										
BRBI	67.6±26.9	66.4±23.2	63.2±27.2	61.5±21.1	0.006*	64.83±24.26	65.30±26.23	63.80±22.06	62.82±22.64	0.741
BRSEF	93.5±13.2	89.1±16.2	88.7±14.6	83.1±18.3	＜0.001*	90.65±14.53	89.86±15.49	76.34±21.85	77.56±15.59	＜0.001*
BRFU	50.0±33.6	51.6±30.4	50.6±32.9	54.1±27.8	0.729	49.94±31.62	52.84±31.51	53.12±30.36	50.00±30.18	0.779
BRSEE	94.1±13.6	89.0±17.4	88.5±18.4	79.7±25.4	＜0.001*	89.70±17.77	89.63±19.31	76.34±26.10	74.36±21.72	＜0.001*
**Symptom scales**										
BRST	23.9±15.3	24.0±15.2	24.5±18.1	26.6±19.0	0.701	25.33±16.25	23.24±17.27	26.04±15.25	33.15±18.14	0.014*
BRBS	12.0±17.3	17.6±20.7	16.1±18.6	24.4±21.2	＜0.001*	17.55±19.68	15.10±19.56	23.44±18.86	27.24±21.15	＜0.001*
BRAS	17.8±20.1	20.5±20.6	20.4±18.0	23.2±19.3	0.017*	20.87±19.73	18.23±18.95	27.43±20.54	26.92±22.26	0.013*
BRHL	40.2±31.5	37.4±29.3	39.6±32.6	36.4±27.2	0.850	40.74±30.04	36.61±30.69	31.18±29.73	46.15±26.79	0.048*

Table S3 Quality of life scores(Mean ± SD) by Annual household income, Disease duration

EORTC Items	Annual household income (CYN)				P	Disease duration, months				P
	＜ 30000	30000-80000	80000-150000	＞150000		≤12	13-36	37-60	≥61	
**Panel A - QLQ-C30 Questionnaire**										
GHS	49.2±16.2	52.3±13.0	59.3±13.7	62.8±12.0	＜0.001*	55.7±13.8	53.4±15.6	50.4±12.8	54.7±15.6	0.007*
**Functional scales**										
PF	74.6±20.5	78.8±14.5	72.6±16.7	66.2±16.9	＜0.001*	74.4±17.0	77.6±16.2	77.9±16.1	71.0±19.6	0.009*
RF	73.7±28.3	81.4±23.9	75.5±26.1	71.9±15.5	0.002*	76.6±24.1	79.8±25.8	77.4±24.4	74.3±28.5	0.237
EF	75.6±20.2	76.4±19.2	70.5±19.6	67.2±18.6	0.001*	72.4±18.3	75.7±20.8	77.5±20.5	71.5±18.7	0.003*
CF	77.0±22.0	79.3±18.2	74.3±18.9	68.7±16.3	0.001*	76.4±17.9	78.8±19.2	78.9±20.4	72.3±21.1	0.013*
SF	65.1±27.6	72.0±24.2	72.7±22.8	63.5±14.9	0.025*	69.7±23.0	70.6±26.0	73.4±23.3	65.3±25.7	0.068
**Symptom scales**										
FA	35.7±21.5	31.7±17.0	35.7±15.3	36.1±18.5	0.004*	34.3±16.2	32.2±19.2	32.3±18.3	38.5±18.2	0.004*
NV	18.8±22.4	16.2±19.8	22.3±22.6	26.6±21.5	0.006*	21.9±20.7	16.4±21.5	14.1±17.5	23.5±24.9	0.001*
DI	6.2±13.5	8.3±17.1	15.7±23.0	24.0±22.8	＜0.001*	13.2±19.9	8.6±17.5	4.8±12.5	14.7±22.9	＜0.001*
FI	42.2±30.7	32.2±27.6	30.3±27.7	37.5±25.0	0.001*	33.7±28.5	35.0±28.5	30.1±26.8	40.4±30.4	0.067
PA	29.9±21.5	27.3±19.8	29.7±18.5	31.8±17.6	0.269	28.5±18.3	27.4±20.6	27.8±20.2	33.2±20.5	0.073

DY	14.7±20.0	14.1±20.8	23.7±26.1	24.0±15.2	＜0.001*	18.6±21.0	13.4±21.5	16.1±21.4	22.9±25.1	0.001*
SL	29.6±24.7	30.1±23.2	34.6±25.6	35.4±25.3	0.180	35.1±23.9	26.1±23.3	32.4±27.0	33.6±22.9	0.001*
AP	21.3±25.4	22.5±23.8	28.3±27.2	30.2±24.5	0.024*	27.9±26.3	19.5±24.0	21.1±24.1	28.7±25.4	0.001*
CO	22.4±26.0	21.7±24.4	29.4±27.8	36.5±30.9	0.003*	28.2±26.8	23.2±24.9	20.5±25.8	25.1±28.0	0.056
**Panel B - QLQ-BR23 Questionnaire**										
**Functional scales**										
BRBI	61.8±29.0	68.3±24.7	64.0±21.2	57.3±18.3	0.001*	63.0±24.2	67.4±25.4	67.0±25.9	61.5±24.2	0.004*
BRSEF	93.5±12.6	91.3±14.6	84.6±17.4	68.7±13.9	＜0.001*	85.4±17.6	92.0±14.4	92.0±13.6	86.7±16.3	＜0.001*
BRFU	45.7±34.9	53.0±31.6	55.1±26.6	50.0±29.3	0.094	50.3±29.6	55.2±31.8	50.0±32.9	48.3±31.9	0.235
BRSEE	93.4±13.8	92.1±15.7	82.3±23.0	61.5±25.5	＜0.001*	83.8±22.8	91.2±16.9	92.3±15.5	86.7±20.3	0.001*
**Symptom scales**										
BRST	26.8±17.4	22.4±15.2	24.6±18.4	33.5±15.7	0.001*	26.2±16.8	21.9±16.5	24.5±16.8	27.4±17.1	0.013*
BRBS	17.4±20.2	11.9±16.3	23.0±21.9	30.2±20.5	＜0.001*	20.4±20.3	13.8±17.7	13.1±19.2	21.5±21.6	＜0.001*
BRAS	23.7±22.3	16.0±17.3	22.4±19.3	26.7±19.1	＜0.001*	20.5±18.4	18.3±19.8	19.5±20.2	23.9±20.5	0.064
BRHL	42.9±34.5	37.1±30.7	36.9±25.0	36.6±26.3	0.459	39.6±29.4	37.1±30.7	36.8±30.7	41.3±30.8	0.444

Table S4 Quality of life scores (Mean ± SD) by Metastatic breast cancer state (State M), Menopausal status

EORTC Items	Metastatic breast cancer state (State M)		P	Menopausal status		P
	No	Yes		Post-menopausal	Pre-menopausal	
**Panel A - QLQ-C30 Questionnaire**						
GHS	55.0±14.7	49.9±13.9	0.001*	51.0±15.6	57.3±12.6	＜0.001*
**Functional scales**						
PF	75.8±16.4	74.5±19.9	0.958	75.7±18.6	75.2±15.4	＜0.001*
RF	78.8±24.6	72.6±28.1	0.021*	76.0±26.9	78.9±23.7	0.001*
EF	74.6±19.1	73.2±21.7	0.703	73.3±20.8	75.4±18.3	0.052
CF	77.8±18.3	74.0±22.9	0.186	75.8±20.8	78.2±17.7	0.012*
SF	71.5±24.1	64.5±25.6	0.006*	69.3±26.1	70.7±22.7	0.118
**Symptom scales**						
FA	33.3±17.2	36.4±20.6	0.260	35.2±19.9	32.6±15.4	0.016*
NV	18.6±20.8	20.2±23.5	0.794	18.3±21.2	19.9±21.8	＜0.001*
DI	10.5±19.0	10.2±18.7	0.920	8.6±18.1	12.6±19.6	0.001*
FI	33.1±28.8	39.7±27.9	0.006*	36.4±29.7	32.5±27.3	0.545

PA	28.0±19.2	31.7±21.7	0.108	31.1±20.9	26.1±18.1	0.001*
DY	16.6±21.5	19.1±24.3	0.357	16.7±22.6	17.9±21.7	＜0.001*
SL	30.7±24.0	33.8±25.5	0.231	31.0±24.5	31.9±24.3	0.225
AP	23.0±24.4	27.7±27.9	0.111	23.6±25.2	24.6±25.4	0.038*
CO	24.5±25.9	25.1±27.9	0.964	24.4±27.0	24.8±25.5	0.136
**Panel B - QLQ-BR23 Questionnaire**						
**Functional scales**						
BRBI	65.3±23.9	63.8±28.2	0.996	62.7±26.7	67.6±22.5	0.090
BRSEF	88.5±16.4	90.4±14.1	0.379	92.1±14.1	85.2±17.2	＜0.001*
BRFU	53.0±30.7	46.3±33.3	0.040*	49.6±32.5	53.8±29.8	0.902
BRSEE	88.1±19.80	89.1±18.9	0.627	91.0±18.3	85.1±20.6	＜0.001*
**Symptom scales**						
BRST	23.9±16.5	27.5±17.7	0.034*	25.3±17.4	24.0±16.2	0.023*
BRBS	16.5±19.0	19.1±22.3	0.414	16.9±20.4	17.4±19.1	＜0.001*
BRAS	18.9±18.7	24.6±21.9	0.017*	21.5±20.6	18.7±18.3	0.001*
BRHL	36.8±29.4	44.4±32.5	0.023*	42.6±31.6	33.6±27.9	0.450
